# Strategy Optimization of Quality Improvement and Price Subsidy of Agri-Foods Supply Chain

**DOI:** 10.3390/foods11121761

**Published:** 2022-06-15

**Authors:** Jing Xu, Jiajia Cai, Guanxin Yao, Panqian Dai

**Affiliations:** 1 Jiangsu Modern Logistics Research Base, Business School, Yangzhou University, Yangzhou 225127, China; caijiajia2910@163.com (J.C.); daipanqian@126.com (P.D.); 2 Research Institute of China Grand Canal, Yangzhou University, Yangzhou 225127, China; gxyao@yzu.edu.cn

**Keywords:** agri-food supply chain, quality safety, price compensation, market demand, cost-sharing contracts

## Abstract

Based on the realistic concerns about the improvement of the quality of agricultural foods (agri-foods), the optimal supply quality and price subsidy strategies of producers and sellers for the two-level agricultural supply chain, composed of a producer and a seller, are studied. The differences in the quality safety, price, and market demand of agri-foods in the supply chain are compared and analyzed. The study found that the maximum profit of supply chain participants decreases with the increase of price elasticity of demand. When the quality of agri-foods is upgraded in a producer-led manner, the quality of agri-foods in the supply chain does not undergo substantial improvement, and the maximum profit of agri-foods operators is insensitive to the price elasticity of demand at this time. When the seller-led quality upgrading is launched, the maximum profit of the producer decreases with the increase of the quality elasticity of demand, the maximum profit of the seller increases with the increase of the quality elasticity of demand, and the total profit of the supply chain also increases with the increase of the quality elasticity of demand under the centralized decision situation. The quality and safety of agri-foods as well as the overall profit of the supply chain can be improved most effectively under the centralized control decision with the goal of maximizing the supply chain benefits. In terms of quality and price, quality improvement actions of agri-foods driven by supply-side producers are less effective than those driven by demand-side consumption. In addition, cost-sharing contracts can significantly improve the quality of agri-foods in the supply chain and make them more “high-quality and low-price” than before the adoption of cost-sharing contracts.

## 1. Introduction

For a long time, the phenomenon that agricultural foods (agri-foods) with good quality do not sell at a good price is common, and the main reason for this is the credence property of agricultural products [1]. Farmers need to invest more in quality to get high-quality agri-foods compared with ordinary produce. However, the quality of the produce cannot be easily identified by consumers. Due to asymmetric information, consumers are not willing to pay high prices for high-quality produces [2]. As a result, agricultural producers often lack the motivation to improve the quality of agri-foods, leading to the prevalence of homogeneous “big-ticket” products in the field of agricultural supply [3].

With the development of economy and society, consumers’ income level and consciousness of food safety are rising, and the consumption demand for agricultural produce is gradually changing from concern for supply quantity to supply quality and diversity [4]. Further, the consumers’ demand for premium quality agri-foods is increasingly strong, which has driven more and more participants in the agricultural supply chain to increase investment for the improvement of agri-foods quality. Some measures such as establishing production standard systems and adopting clean production technologies are used. After taking the quality improvement actions, agricultural operators apply for pollution-free, green, or organic certification as a way to increase the premium price of their produce and build agricultural brands. This information is ultimately passed on to the consumer in the form of labels on the produce [5,6,7]. Nowadays, in the field of practice, this quality improvement measure has been launched either by agricultural producers on their own initiative or by sellers who induce agricultural producers to improve their quality inputs in the form of high-price subscriptions [8]. However, there is still no relevant theory to guide the practice of which model is better. Therefore, which way is more beneficial to achieve quality and price of agri-foods? How do the participants in the agricultural supply chain make quality improvement and price subsidy decisions? Additionally, can appropriate contracts be designed to encourage producers and sellers to increase their investment in agricultural quality improvement actions? With these questions, we conducted this study.

## 2. Literature Review

### 2.1. The Improvement Strategy of Product Quality

A review of the literature revealed that how to improve product quality to enhance market competitiveness has been a hot research topic in business operation management. For example, Li et al. studied the supplier’s product upgrade strategies considering consumers’ strategic behavior [9]. Chen et al. studied the joint pricing and quality decision problems in a dual-channel supply chain [10]. Li et al. studied pricing and quality competition issues under brand differentiation [11]. These studies focus on consumer reactions to product quality upgrades in a highly competitive context. Unlike general products, the problem of structural imbalance on the supply side of agricultural produce has existed for many years. In other words, the oversupply of general mass agri-foods and the undersupply of high-quality agri-foods coexist. In addition, agricultural producers are mostly in a disadvantaged position, operating at low profit. Under the urgent call of consumers for high-quality and safe agri-foods, even if they make quality improvement behaviors, they need the supply chain to bear the cost of quality improvement inputs and put produce into the market with high quality and competitive price. Otherwise, the agricultural supply chain lacks the incentive for quality improvement. Although scholars also pay attention to the quality improvement of agricultural products, they mainly focus on the quality grading [12,13], quality and safety regulation [14], and related behaviors affecting the quality and safety of agricultural produce [15]. Moreover, data surveys and empirical tests are mainly used [16,17], which cannot effectively predict the long-term impact of agri-foods quality upgrading.

### 2.2. Game Study on Product Price and Demand

In terms of supply chain games considering price and demand, Abad [18] constructed a demand–price-sensitive supply chain coordination model and finally obtained a method to achieve the optimal strategy when buyers and sellers cooperate. Sajadi et al. discussed the issue of supply chain coordination when market demand is influenced by factors such as the size of the retailer’s effort to develop the market and the retail price [19]. Chan and Dai et al. discussed the problems of optimal order lot size and optimal order lead time for achieving maximum profit in the supply chain in a cooperative game, respectively [20,21]. Mir Mehdi et al. constructed non-cooperative game models, such as the Nash game and the Stackelberg game, as well as a cooperative game model to study a two-level supply chain game problem in which demand is influenced by advertising and price [22]. Zhao et al. constructed an option contract model to solve the conflict of interest in the supply chain [23]. Leng et al. used the Nash and Stackelberg game model to study the multi-supplier but single-retailer supply chain coordination problem for short life-cycle products [24]. Esmaeili et al. studied the supply chain game problem when demand is dependent on price and marketing costs [25]. In general, the means and game methods of supply chain coordination under different conditions have been studied extensively and thoroughly, but not much research has been conducted on quality and safety levels of agri-foods and corresponding price compensation mechanisms. For instance, Ren et al. studied the performance of food safety management systems of Chinese food business operators [26]. Jacques et al. [27] discussed the quality and safety standards in the food industry. Though the quality of agri-foods is noticed, there is no mention of subsidies for the implementors of the agricultural produce improvement initiative, making it difficult to provide targeted guidance for quality improvement and cost–price compensation in agricultural supply chains. Therefore, we conducted the present study based on our previous research on the quality [2] and price compensation [28] mechanisms of agricultural produce.

### 2.3. Influences after the Improvement of the Agri-Food Quality

Some other researchers discussed the influence of the foods label that reflects the quality of produce. Messer et al. proposed that labeling produce can have both good and ugly effects [5]. The good effects include that the labeling of quality can reduce information asymmetry, and the ugly effects includes that the labeling and certifying process may be costly, which leads to reduced demand for high-quality agricultural produces. Aftab et al. analyzed the impact of rising food prices on consumer welfare in the most populous countries of South Asia and found that consumer welfare declines in all countries mainly for cereals and milk, as these food items are relatively less elastic to price fluctuations [29]. The labeling process and price increases associated with improved quality of agricultural produce will undoubtedly have an impact on social welfare and other stakeholders. However, this is not the focus of this paper. Different from these articles, we only focused on the quality improvement action though it is a key part of the produce before it is labeled. In addition, the quality improvement studied in this article is voluntary and endogenous, proposed by the participants of the agricultural supply chain. As a result, in order to focus more on the research topic, we will not discuss at great length the social impact of quality improvement actions.

Above all, compared to existing research, the novelty of our work is as follows: The “quality safety factor” and “price compensation factor” are introduced into the linear inverse demand function of agricultural produce for the first time. By constructing three different game models under decentralized and centralized decision-making situations, the optimal strategy for upgrading the quality of agricultural produce is analyzed, and a cost-sharing contract is designed to coordinate the supply chain. In this way, some theoretical reference is provided for the participants in the agricultural supply chain when making decisions about the quality improvement input and price compensation.

## 3. Materials and Methods

### 3.1. Problem Description

A two-level agricultural supply chain consisting of a risk-neutral agri-foods producer and a risk-neutral agri-foods seller is studied in this paper. Prior to the marketing season, the seller orders q quantities of common produce from the produce producer at a price of $p_{0}$. However, considering that market demand is influenced by both the quality level and retail price of agricultural produce, and more and more consumers prefer green and organic agri-foods, the “Agricultural Produce Quality and Safety Enhancement Initiative” is proposed for the agricultural supply chain. This initiative aims at encouraging agricultural producers to increase quality inputs to improve the quality of agri-foods. Thus, the agricultural produce is as follows:

(1) In order to respond to the quality and safety enhancement initiative, the agricultural producer needs to increase the quality inputs, such as upgrading production technology level, improving production management methods, etc. Referring to the study of Jiang [30], the research and development inputs for producing green products have a quadratic relationship with the greenness of the products. Therefore, it is assumed that the relationship between quality inputs u and quality safety degree g in agricultural production is $u=\frac{1}{2}zg^{2}$*,* where z is the production influence factor.
(2) When the quality improvement action is promoted, the agricultural producer will raise the price to maintain his own interests according to the input costs of quality improvement. At this point, the selling price of the producer is $w=(1+\alpha \;)w_{0}$, where α is the price compensation coefficient after quality inputs. In order to encourage quality improvement and maintain profits, the agri-foods seller will also raise prices accordingly. To simplify the model, it can be assumed that the seller of agricultural produce raises prices by the same amount as the producer, and at this time, the selling price of the seller is $p=(1+\alpha \;)p_{0}$.
(3) Although the seller of agricultural produce determines the basic market demand q based on past experience and order from the producer, natural losses such as bumping and spoilage cannot be avoided in the actual transportation and sales process due to the perishable nature of agri-foods. Thus, it is assumed that the actual market demand is d (d<q); i.e., the single-order quantity of the seller is greater than the actual market demand. Since agri-foods are perishable, it is assumed that all agri-foods unsold are lost and no longer converted into value.
(4) Assume that the actual market demand d is positively related to the basic market demand q and the quality safety degree g and negatively related to the retail price p. Referring to the linear inverse demand function, let $d=q-b(1+\alpha \;)p_{0}+kg$, where b is the sensitivity coefficient of consumers to the price of agri-foods, and *k* is the sensitivity coefficient of consumers to the quality of agri-foods. Obviously, the higher the product quality is, the lower the price is, and the more consumers prefer to buy such produce. This assumption is in line with the reality.

### 3.2. Notation

Based on the problem description and assumptions above, the notations used in this study are described in Table 1 below.

## 4. Model Construction and Solving

### 4.1. Decentralized Decision Model

According to the above assumptions, the profit function of agricultural producer after implementing quality improvement actions is

(1) $$\pi_{n}=q\left(\left(1+\alpha \right)w_{0}-c_{n}-\frac{1}{2}zg^{2}\right),$$

The profit function of the agricultural produce seller is

(2) $$\pi_{s}=p_{0}\left(1+\alpha \right)\left(q-\;bp_{0}\left(1+\alpha \right)+\mathrm{kg}\right)-q\left(\left(1+\alpha \right)w_{0}+c_{s}\right),$$

**Proposition** **1.** *There exists a quality safety degree* g *that maximizes the profit of the agricultural producer and a level of price subsidy* α *that maximizes the profit of the agri-foods seller.*

**Proof.**  The profit function $\;\pi_{n}$ is as follows:

$$\pi_{n}=q\left[\left(1+\alpha \right)w_{0}-c_{n}-\frac{1}{2}zg^{2}\right]$$

The partial derivative of $\pi_{n}$ with respect to g yields $\frac{\partial \pi_{n}}{\partial g}=-qzg\neq 0$, $\frac{\partial^{2}\pi_{n}}{\partial^{2}g}=-qz<0$, (q>0, z>0). Therefore, $\pi_{n}\;$ is a strictly concave function on *g*; i.e., there exists a maximum value of the profit function $\pi_{n}$.  □

The profit function $\;\pi_{s}$ is as follows:

$$\pi_{s}=p_{0}\left(1+\alpha \right)\left(q\;-\;bp_{0}\left(1+\alpha \right)+\mathrm{kg}\right)-\;q\left(\left(1+\alpha \right)w_{0}+c_{s}\right)$$

The partial derivative of $\pi_{s}$ with respect to α yields $\frac{\partial \pi_{s}}{\partial \alpha }=-2\left(1+\alpha \right)bp_{0}{}^{2}+\left(kg+q\right)p_{0}-qw_{0}\neq 0$, $\frac{\partial^{2}\pi_{s}}{\partial^{2}\alpha }=-bp_{0}{}^{2}<0$. Therefore, $\pi_{s}\;$ is a strictly concave function on α; i.e., there exists a maximum value of the profit function $\pi_{s}$.

(1) Producer-led Model (Model 1)

In this case, the producer is the leader of the supply chain, and the seller is the follower, such as the family farm-dominated or cooperative-dominated agricultural supply chain. Hence, this is a Stackelberg game model dominated by agricultural producer. In this model, the producer determines the quality input u (or quality safety degree g), and then, the seller chooses the optimal price compensation factor $\alpha^{*}$ based on the producer’s input, and the backward induction method is applied to solve the model.

When $\frac{\partial \pi_{n}}{\partial g}=-qzg=0$, the optimal quality safety degree can be obtained as follows:

(3) $$g_{1}^{*}=0,$$

Substitute $g_{1}^{*}$ into Equation (1); when $\frac{\partial \pi_{s}}{\partial \alpha }=p_{0}q-2bp_{0}{}^{2}\alpha -2bp_{0}{}^{2}-qw_{0}=0$, the optimal price subsidy factor can be obtained as follows:

(4) $$\alpha_{1}^{*}=\frac{q(p_{0}-w_{0})}{2bp_{0}^{2}}-1,$$

Obviously, the agricultural producer as the leader lacks the incentive to improve the quality in this case, but the seller still provides price subsidies due to information asymmetry. The market demand is $d_{1}^{*}=\frac{q(p_{0}+w_{0})}{2p_{0}}$. Substituting Equations (3) and (4) into Equations (1) and (2), the optimal profit of the agricultural producer and the optimal profit of the agri-foods seller are obtained as follows:

$$\pi_{n}^{1*}=\frac{q^{2}(p_{0}-w_{0})w_{0}}{2bp_{0}^{2}}-qc_{n}$$

$$\pi_{s}^{1*}=\frac{q^{2}p_{0}^{2}-2q^{2}p_{0}w_{0}+q^{2}w_{0}^{2}}{4bp_{0}^{2}}-qc_{s}$$

**Proposition** **2.** *When decentralized decision making is dominated by the agricultural producer, the maximum profit of both producer and seller decreases with the increase of price elasticity of consumer demand, and the maximum profit of the agricultural seller increases as the retail price per unit of produce increases. When* $p_{0}<2w_{0}$ *, the maximum profit of the agricultural producer increases with the increase of the retail price per unit of agricultural produce. Likewise, when* $p_{0}>2w_{0}$ *, the maximum profit of the agricultural producer decreases with the increase of the retail price per unit of agri-foods.*

**Proof.**  From the equations $\frac{\partial \pi_{n}^{1*}}{\partial b}=-\frac{\left(p_{0}-w_{0}\right)w_{0}q^{2}}{2p_{0}^{2}b^{2}}<0$, $\frac{\partial \pi_{s}^{1*}}{\partial b}=-\frac{{\left(p_{0}-w_{0}\right)}^{2}q^{2}}{4p_{0}^{2}b^{2}}<0$, and $\frac{\partial \pi_{s}^{1*}}{\partial p_{0}}=\frac{\left(p_{0}-w_{0}\right)w_{0}q^{2}}{2p_{0}^{3}b}>0$, it can be obtained that $\pi_{n}^{1*}$, $\pi_{s}^{1*}$ are negatively correlated with b, while $\pi_{s}^{1*}$ is positively correlated with $p_{0}$. When $p_{0}<2w_{0}$, $\frac{\partial \pi_{n}^{1*}}{\partial p_{0}}>0$ can be known, and otherwise,$\;\frac{\partial \pi_{n}^{1*}}{\partial p_{0}}<0$.  □

Proposition 2 shows that the maximum profit of agricultural supply chain participants is closely related to the level of economic and social development and the consumption environment. When the income level of consumers is low (high price elasticity of demand), the profit of agricultural operators is also slimmer. The higher the price of the produce before quality improvement, the higher the profit for the produce seller to participate in quality improvement. While the profit of agricultural producers is mainly influenced by the level of their price appreciation in the supply chain before quality improvement, if the retail price of agri-foods exceeds the wholesale price by more than two times, the lower the retail price of agri-foods before the implementation of quality improvement actions, and the greater the producer’s profit.

(2) Seller-led Model (Model 2)

In this case, the seller of agri-foods is the leader of the supply chain, while the producer is the follower, such as the “company + farmer” type of agricultural supply chain. Therefore, this is a Stackelberg game model dominated by agricultural seller. The price subsidy coefficient α is determined by the seller of agri-foods firstly, and then, the ideal quality safety degree $g^{*}$ and the optimal quality input level are determined by the producer according to α, and the backward induction method is also used to solve the model.

When $\frac{\partial \pi_{s}}{\partial \alpha }=p_{0}q+p_{0}kg-2bp_{0}{}^{2}\alpha -2bp_{0}{}^{2}-qw_{0}=0$, the optimal price subsidy system can be obtained as follows: $\alpha_{2}^{*}\left(g\right)=\frac{p_{0}q+kgp_{0}-w_{0}q}{2bp_{0}^{2}}-1$. Substitute $\alpha_{2}^{*}\left(g\right)$ into Equation (2); when $\frac{\partial \pi_{n}}{\partial g}=\frac{kw_{0}}{2bp_{0}}-zg=0$, the optimal quality safety degree can be obtained as follows:

(5) $$g_{2}^{*}=\frac{kw_{0}}{2zbp_{0}},$$

Substitute Equation (5) into the optimal response function $\alpha_{2}^{*}\left(g\right)$ of the produce seller, and then, obtain the optimal price subsidy level for the quality improvement of agri-foods as follows:

(6) $$\alpha_{2}^{*}=\frac{qzb\left(p_{0}-w_{0}\right)+0.5k^{2}w_{0}}{2zb^{2}p_{0}^{2}}-1,$$

Thus, the actual market demand is $d_{2}^{*}=\frac{qzb\left(p_{0}+w_{0}\right)+0.5k^{2}w_{0}}{2\mathrm{zb}p_{0}}$. Based on this, substituting Equations (5) and (6) into Equations (1) and (2), the optimal profit of the agricultural producer and the optimal profit of the agricultural seller are obtained as follows:

$$\pi_{n}^{2*}=\frac{\left(bqzp_{0}w_{0}-qzbw_{0}^{2}-0.25k^{2}w_{0}^{2}\right)q}{2zb^{2}p_{0}^{2}}-qc_{n}$$

$$\pi_{s}^{2*}=\frac{q^{2}z^{2}{\left(p_{0}-w_{0}\right)}^{2}b^{2}+k^{2}qzbw_{0}\left(p_{0}-w_{0}\right)+0.25k^{4}w_{0}^{2}}{4z^{2}b^{3}p_{0}^{2}}-qc_{s}$$

**Proposition** **3.** *When decentralized decision making is dominated by sellers of agri-foods, the maximum profit of both agricultural producer and seller decreases with the increase of production impact factor* z *. The maximum profit of agricultural producer decreases as the quality elasticity of demand increases, and the price elasticity of demand decreases. The maximum profit of agricultural seller increases as the quality elasticity of demand increases, and the price elasticity of demand decreases.*

**Proof.**  From the equations $\frac{\partial \pi_{n}^{2*}}{\partial k}=-\frac{qkw_{0}^{2}}{4zp_{0}^{2}b^{2}}<0$, $\frac{\partial \pi_{n}^{2*}}{\partial z}=-\frac{qk^{2}w_{0}^{2}}{8z^{2}p_{0}^{2}b^{2}}<0$, and $\frac{\partial \pi_{n}^{2*}}{\partial b}=-\frac{q^{2}zbw_{0}\left(w_{0}-p_{0}\right)-0.25qk^{2}w_{0}^{2}}{2zp_{0}^{2}b^{3}}>0$, it can be obtained that $\pi_{n}^{2*}$ is negatively correlated with k and z and positively correlated with b. Additionally, $\pi_{s}^{2*}$ is positively correlated with k and negatively correlated with z, and b can be known due to $\frac{\partial \pi_{s}^{2*}}{\partial k}=\frac{kqzbw_{0}\left(p_{0}-w_{0}\right)+0.5w_{0}^{2}k^{3}}{2z^{2}p_{0}^{2}b^{3}}>0$, $\frac{\partial \pi_{s}^{2*}}{\partial z}=-\frac{k^{2}w_{0}\left(qbz\left(p_{0}-w_{0}\right)+0.5k^{2}w_{0}\right)}{4z^{3}p_{0}^{2}b^{3}}<0$, and $\frac{\partial \pi_{s}^{2*}}{\partial b}=\frac{-q^{2}z^{2}b^{2}{\left(p_{0}-w_{0}\right)}^{2}-2qzbk^{2}w_{0}\left(p_{0}-w_{0}\right)-0.75k^{4}w_{0}^{2}}{4z^{2}p_{0}^{2}b^{4}}<0$.  □

Proposition 3 shows that the greater the quality elasticity of consumer demand, the less price sensitive consumers are; and the smaller the quality inputs required to improve the quality and safety of agricultural products, the greater the maximum profit for agricultural sellers is. When consumers pay more attention to quality, the supply chain profit distribution will be more unfavorable to producers, which will then force the producer to improve quality input, and the producer’s cost increases, and the profit decreases in a short period of time.

### 4.2. Centralized Decision Model (Model 3)

In this case, self-interest maximization is no longer the decision-making goal of the participants of agricultural supply chain. Instead, centralized decision making is made through win-win cooperation to maximize the overall interests of the supply chain. At this time, the total profit function of the agricultural supply chain is:

$$\pi_{ns}=p_{0}\left(1+\alpha \right)\left(q-bp_{0}\left(1+\alpha \right)+\mathrm{kg}\right)-q\left(\left(1+\alpha \right)w_{0}+c_{s}\right)+q\left(\left(1+\alpha \right)w_{0}\;-c_{n}-\frac{1}{2}zg^{2}\right)$$

**Proposition** **4.** *When*$2bqz-k^{2}>0$, $\pi_{ns}$*is concave in*α*andg. In this case, the overall profit function of the supply chain has a maximum value.*

**Proof.**  The Hessian matrix of $\pi_{\mathrm{ns}}$ is $\left|\begin{array}{c} \frac{\partial^{2}\pi_{\mathrm{ns}}}{\partial^{2}g}\\ \frac{\partial^{2}\pi_{\mathrm{ns}}}{\partial \alpha \partial g} \end{array}\;\begin{array}{c} \frac{\partial^{2}\pi_{\mathrm{ns}}}{\partial g\partial \alpha }\\ \frac{\partial^{2}\pi_{\mathrm{ns}}}{\partial^{2}\alpha } \end{array}\right|=\left|\begin{array}{c} -qz\\ kp_{0} \end{array}\;\begin{array}{c} kp_{0}\\ -2bp_{0}^{2} \end{array}\right|=\left(2bqz-k^{2}\right)p_{0}^{2}$. When $2bqz-k^{2}>0,$ conditions $\left(2bqz-k^{2}\right)p_{0}^{2}>0$ and −qz<0 can be satisfied, so the Hessian matrix is negative definite. The profit function $\pi_{ns}$ is a joint concave function with respect to the price subsidy coefficient α and the quality safety degree g. Hence, there exists optimal solutions $\alpha_{3}^{*}$ and $g_{3}^{*}$ to maximize the profit function.  □

By combining the equations $\frac{\partial \pi_{ns}}{\partial \alpha }=p_{0}q+p_{0}kg-2bp_{0}^{2}-2bp_{0}^{2}\;\alpha =0$ and $\frac{d\pi_{ns}}{dg}=p_{0}k+p_{0}k\alpha -qzg=0$, the optimal price subsidy coefficient and quality safety degree are obtained as follows:

$$\alpha_{3}^{*}=\frac{zq^{2}}{p_{0}\left(2bqz-k^{2}\right)}-1$$

$$g_{3}^{*}=\frac{qk}{2bqz-k^{2}}$$

At this point, the actual market demand is $d_{3}^{*}=\frac{bzq^{2}}{2bqz-k^{2}}$. The maximum profit of the agricultural supply chain under centralized decision making is

$$\pi_{\mathrm{ns}}{}^{*}=\frac{0.5zq^{3}}{2bqz-k^{2}}-\left(c_{s}+c_{n}\right)q$$

**Proposition** **5.** *In the centralized model, the overall profit of the agricultural supply chain increases with the increase of the quality elasticity of demand* k *and decreases with the increase of the price elasticity of demand* b *and the production impact factor* z *. That is, when consumers are more concerned about quality and less concerned about price, and the smaller the cost of quality inputs required by agricultural producers to implement quality improvement actions, the greater the overall profitability of the supply chain.*

**Proof.**  Due to $\frac{\partial \pi_{\mathrm{ns}}{}^{*}}{\partial b}=-\frac{q^{4}z^{2}}{{\left(2qzbp_{0}-k^{2}\right)}^{2}}<0$, $\frac{\partial \pi_{\mathrm{ns}}{}^{*}}{\partial k}=\frac{kzq^{3}}{{\left(2qzb-k^{2}\right)}^{2}}>0$, and $\frac{\partial \pi_{\mathrm{ns}}{}^{*}}{\partial z}=-\frac{0.5k^{2}q^{3}}{{\left(2qzb-k^{2}\right)}^{2}}<0$, $\pi_{\mathrm{ns}}{}^{*}$ is positively correlated with k and negatively correlated with b and z.  □

### 4.3. Comparison and Analysis

According to the calculation results above, summarizing the quality improvement and price subsidy decisions of the agricultural supply chain participants as well as the corresponding changes in market demand, Table 2 can be obtained below.

The following prerequisites can be derived from the results in Table 2:

(1) From $\alpha_{1}{}^{*}=\frac{q(p_{0}-w_{0})}{2bp_{0}^{2}}-1>0$, $q\;\left(p_{0}-w_{0}\right)>2bp_{0}^{2}$ can be easily obtained.
(2) From $\;\alpha_{3}{}^{*}=\frac{zq^{2}}{p_{0}\left(2bqz-k^{2}\right)}-1>0$ and $g_{3}{}^{*}=\frac{qk}{2bqz-k^{2}}>0$,$\;2bqz>k^{2}>2bqz-\frac{zq^{2}}{p_{0}}$ can be obtained.

Based on the discussion above, the following propositions can be known:

**Proposition** **6.** *The quality of agri-foods in the centralized decision model is higher than that in the decentralized model when the participants of the agricultural supply chain make decisions to maximize the profit of the supply chain. In addition, the quality of agri-foods is higher when the seller is the leader than in the producer-led case. That is, the best quality safety degree satisfies*$g_{1}{}^{*}<g_{2}{}^{*}<g_{3}{}^{*}$.

**Proof.**  Due to $g_{2}{}^{*}=\frac{kw_{0}}{2zbp_{0}}$, and since k, $w_{0}$, z, b, $p_{0}$ are positive, $g_{2}{}^{*}>g_{1}{}^{*}=0$ is satisfied. Again, due to $g_{3}{}^{*}=\frac{qk}{2bqz-k^{2}}$, for $g_{3}{}^{*}>g_{2}{}^{*}$ to be true, condition $2bqzk\left(p_{0}-w_{0}\right)+k^{3}w_{0}>0$ should be satisfied. Since the selling price is greater than the buying price, i.e., $p_{0}-w_{0}>0$, and $k,\;w_{0}$ are positive, $g_{3}{}^{*}>g_{2}{}^{*}$ can be known. That is, $g_{1}{}^{*}<g_{2}{}^{*}<g_{3}{}^{*}$ is proven.  □

**Proposition** **7.** *In general, the optimal price compensation factor satisfies*$\alpha_{1}{}^{*}<\alpha_{2}{}^{*}$and$\alpha_{1}{}^{*}<\alpha_{3}{}^{*}$.

**Proof.**  Due to $\alpha_{1}{}^{*}=\frac{q(p_{0}-w_{0})}{2bp_{0}^{2}}-1$ and $\alpha_{2}{}^{*}=\frac{qzb\left(p_{0}-w_{0}\right)+0.5k^{2}w_{0}}{2zb^{2}p_{0}^{2}}-1$, it can be obtained that $\alpha_{2}{}^{*}-\alpha_{1}{}^{*}=\frac{w_{0}k^{2}}{4zb^{2}p_{0}^{2}}>0$. That is, $\alpha_{1}{}^{*}<\alpha_{2}{}^{*}$. Again, due to $\alpha_{3}{}^{*}=\frac{zq^{2}}{p_{0}\left(2bqz-k^{2}\right)}-1$, for $\alpha_{1}{}^{*}<\alpha_{3}{}^{*}$ to be true, condition $k^{2}\left(w_{0}-p_{0}\right)<2bzqw_{0}$ should be satisfied. Because $w_{0}-p_{0}<0$, i.e., $k^{2}\left(w_{0}-p_{0}\right)<0$, as well as $2bzqw_{0}>0$, $\alpha_{1}{}^{*}<\alpha_{3}{}^{*}$ can therefore be proven.  □

Proposition 7 shows that in this agricultural supply chain, the price compensation factors both in the Stackelberg model dominated by sellers and the centralized decision model are greater than those in the Stackelberg game model dominated by agricultural producers. That is, in terms of quality and price, the quality improvement actions driven by producers on the supply side are not as effective as the quality improvement actions driven by consumption on the demand side.

**Corollary** **1.** *When the initial price of agri-foods*$p_{0}>\frac{k^{2}}{4b^{2}z}$*, the price appreciation of agri-foods satisfies*$\alpha_{1}{}^{*}<\alpha_{2}{}^{*}<\alpha_{3}{}^{*}$.

**Proof.**  For $\alpha_{3}{}^{*}>\alpha_{2}{}^{*}$ to be true, condition $\frac{zq^{2}}{p_{0}\left(2bqz-k^{2}\right)}>\frac{qzb\left(p_{0}-w_{0}\right)+0.5k^{2}w_{0}}{2zb^{2}p_{0}^{2}}$ should be satisfied, which is equivalent to proving that

(7) $$0.5k^{2}w_{0}\left(2bqz-k^{2}\right)-2z^{2}b^{2}q^{2}w_{0}+bqzk^{2}\left(w_{0}-p_{0}\right)<0$$

Firstly, $bqzk^{2}\left(w_{0}-p_{0}\right)<0$ can be easily known, so to prove (7) is true, that is, the proof of $0.5k^{2}w_{0}\left(2bqz-k^{2}\right)-2z^{2}b^{2}q^{2}w_{0}<0$ is satisfied. Secondly, it is clear from the precondition that $\frac{zq^{2}}{p_{0}}>2bqz-k^{2}$, so to prove $0.5k^{2}w_{0}\left(2bqz-k^{2}\right)-2z^{2}b^{2}q^{2}w_{0}<0.5k^{2}w_{0}\frac{zq^{2}}{p_{0}}-2z^{2}b^{2}q^{2}w_{0}$ is as same as to prove $zq^{2}k^{2}w_{0}-4z^{2}b^{2}q^{2}p_{0}w_{0}<0$, that is, to prove that $zw_{0}q^{2}\left(k^{2}-4zb^{2}p_{0}\right)<0$, which means $p_{0}>\frac{k^{2}}{4b^{2}z}$. Hence, when $p_{0}>\frac{k^{2}}{4b^{2}z}$ is satisfied, $\alpha_{2}{}^{*}<\alpha_{3}{}^{*}$ is true, and $\alpha_{1}{}^{*}<\alpha_{2}{}^{*}<\alpha_{3}{}^{*}$ can be obtained finally.  □

Corollary 1 shows that the price compensation factor in the centralized decision model is greater than the price compensation factor under the decentralized decision model dominated by sellers only when the market price of agri-foods is high, and the condition $p_{0}>\frac{k^{2}}{4b^{2}z}$ is met. Combined with Proposition 6, it can be seen that for high-priced agri-foods, supply chain cooperation to improve the quality of agri-foods is most beneficial for price appreciation, and the quality and price of agri-foods can be realized to the greatest extent.

**Corollary** **2.** *The actual market demand satisfies*$d_{2}{}^{*}>d_{1}{}^{*}$. When$0<w_{0}<\frac{k^{2}p_{0}}{2bzq-k^{2}}$, $d_{3}{}^{*}>d_{1}{}^{*}$, and when$w_{0}>\frac{k^{2}p_{0}}{2bzq-k^{2}}$*,*$d_{3}{}^{*}<d_{1}{}^{*}$. When$0<w_{0}<\frac{2bzqk^{2}p_{0}}{4b^{2}z^{2}q^{2}-k^{4}}$*,*$d_{3}{}^{*}>d_{2}{}^{*}$, and when$w_{0}>\frac{k^{2}p_{0}}{2bzq-k^{2}}$*,*$d_{3}{}^{*}<d_{2}{}^{*}$.

**Proof.**  Due to $d_{1}{}^{*}=\frac{q\left(p_{0}+w_{0}\right)}{2p_{0}}$ and $d_{2}{}^{*}=\frac{qzb\left(p_{0}+w_{0}\right)+0.5k^{2}w_{0}}{2zbp_{0}}$, $d_{2}{}^{*}-d_{1}{}^{*}=\frac{w_{0}k^{2}}{4zbp_{0}}>0,$ which means $d_{2}{}^{*}>d_{1}{}^{*}$. Again, due to $d_{3}{}^{*}-d_{1}{}^{*}=\frac{0.5\left(w_{0}+p_{0}\right)qk^{2}-bzq^{2}w_{0}}{p_{0}\left(2bzq-k^{2}\right)}$, $0.5\left(w_{0}+p_{0}\right)qk^{2}-bzq^{2}w_{0}<0$ can be known, and then,$\;d_{3}{}^{*}<d_{1}{}^{*}$ is obtained. Since the condition $0.5\left(w_{0}+p_{0}\right)qk^{2}-bzq^{2}w_{0}<0$ is equivalent to $w_{0}>\frac{k^{2}p_{0}}{2bzq-k^{2}}$, when $0<w_{0}<\frac{k^{2}p_{0}}{2bzq-k^{2}}$, $d_{3}{}^{*}>d_{1}{}^{*}$ can be obtained, and when $w_{0}>\frac{k^{2}p_{0}}{2bzq-k^{2}}$, $d_{3}{}^{*}<d_{1}{}^{*}$ can be obtained.  □

Similarly, from the equation $d_{3}{}^{*}-d_{2}{}^{*}=\frac{0.5bzqp_{0}k^{2}-b^{2}z^{2}q^{2}w_{0}+0.25w_{0}k^{4}}{bzp_{0}\left(2bzq-k^{2}\right)}$, $0.5bzqp_{0}k^{2}-b^{2}z^{2}q^{2}w_{0}+0.25w_{0}k^{4}<0$ can be known, which proves $d_{3}{}^{*}<d_{2}{}^{*}$. Since the condition $0.5bzqp_{0}k^{2}-b^{2}z^{2}q^{2}w_{0}+0.25w_{0}k^{4}<0$ is equivalent to $w_{0}>\frac{2bzqk^{2}p_{0}}{4b^{2}z^{2}q^{2}-k^{4}}$, $d_{3}{}^{*}>d_{2}{}^{*}$ can be obtained when $0<w_{0}<\frac{2bzqk^{2}p_{0}}{4b^{2}z^{2}q^{2}-k^{4}}$, and $d_{3}{}^{*}<d_{2}{}^{*}$ can also be obtained when $w_{0}>\frac{k^{2}p_{0}}{2bzq-k^{2}}$.

Corollary 2 shows that in case of decentralized models, a seller-led initiative to improve the quality of agri-foods is more beneficial to increase actual market sales than a producer-led one. The centralized decision is more favorable to increase the sales volume of agri-foods when the agricultural producers sell to sellers with low unit price. Furthermore, the actual market demand in the centralized model is less than that in the decentralized decision case when the agricultural producers sell to sellers with high-value agri-foods.

## 5. Contract Coordination Strategy Based on Cost Sharing of Agricultural Quality Improvement

According to the analysis above, the quality improvement of agri-foods driven by the demand side is more effective. Based on this, referring to the cost-sharing contract model of Yang et al. [31], it is assumed that agri-foods sellers are willing to share the quality improvement cost at a ratio of $\beta \in \left(0,1\right)$, which incentivizes producers to increase inputs on agri-food quality improvement. In the decentralized scenario dominated by sellers, the profit functions of the agricultural producers and sellers are

$$\pi_{n}=q\left(\left(1+\alpha \right)w_{0}-c_{n}-\left(1-\beta \right)\frac{1}{2}zg^{2}\right)$$

$$\pi_{s}=p_{0}\left(1+\alpha \right)\left(q-bp_{0}\left(1+\alpha \right)+kg\right)-q\left(\left(1+\alpha \right)w_{0}+c_{s}+\frac{1}{2}\beta zg^{2}\right)$$

Extending the solution method in Model 2, the optimal decisions and profits of producers and sellers of agri-foods under cost-sharing contracts can be obtained as follows:

$$g_{d}^{*}=\frac{0.5kw_{0}}{\left(1-\beta \right)zbp_{0}}$$

$$\alpha_{d}^{*}=\frac{0.25k^{2}w_{0}}{zb^{2}p_{0}{}^{2}\left(1-\beta \right)}-1$$

At this point, the profits of the participants in the agricultural supply chain are:

$$\pi_{n}^{d*}=\frac{0.5k^{2}w_{0}{}^{2}q}{4zb^{2}p_{0}{}^{2}\left(1-\beta \right)}-qc_{n}$$

$$\pi_{s}^{d*}=\frac{\left(\left(1-\beta \right)p_{0}+\left(0.5\beta -1\right)w_{0}\right)w_{0}k^{2}qzb+0.25w_{0}{}^{2}k^{4}}{4b^{3}p_{0}{}^{2}{\left(1-\beta \right)}^{2}z^{2}}-qc_{s}$$

**Proposition** **8.** *The effect of cost contract coordination strategy of this agricultural supply chain depends on the original parameter. When* $2qbzw_{0}=qbzp_{0}+0.75w_{0}k^{2}$ *, the optimal cost-sharing coefficient* $\beta^{*}$ *can be obtained.*

**Proof.**  The second-order derivative of $\pi_{s}{}^{*}$ with respect to β can be obtained as follows:$\;\frac{\partial^{2}\pi_{s}{}^{*}}{\partial^{2}\beta }=\frac{k^{2}w_{0}\left(-qbz\beta p_{0}+qbzp_{0}+0.5qbz\beta w_{0}-2qbzw_{0}+0.75w_{0}k^{2}\right)}{2b^{3}p_{0}{}^{2}{\left(\beta -1\right)}^{4}z^{2}}$. Considering the numerator $-qbz\beta p_{0}+qbzp_{0}+0.5qbz\beta w_{0}-2qbzw_{0}+0.75w_{0}k^{2}$ as $F\left(\beta \right)$, $\frac{\partial^{\prime }F\left(\beta \right)}{\partial^{\prime }\beta }=-0.5qbz\left(p_{0}-0.5w_{0}\right)w_{0}k^{2}<0$ can thus be obtained easily, which indicates $F\left(\beta \right)$ is a decreasing function about $\beta \in \left(0,1\right)$. When β=0, $\mathrm{MaxF}\left(0\right)=qbzp_{0}-2qbzw_{0}+0.75w_{0}k^{2}$ can be known. When $\mathrm{MaxF}\left(0\right)=0$,$qbz\left(2w_{0}-p_{0}\right)=0.75w_{0}k^{2}$ can be obtained. Obviously, when $qbz\left(2w_{0}-p_{0}\right)=0.75w_{0}k^{2}$ holds, if β>0, then $F\left(\beta \right)<F\left(0\right)=0$, at which point $\frac{\partial^{2}\pi_{s}{}^{*}}{\partial^{2}\beta }<0$; i.e., there exists the optimal cost-sharing coefficient that makes $\pi_{s}{}^{*}$ maximize. Moreover, $\beta^{*}=\frac{qbz\left(2p_{0}-3w_{0}\right)+k^{2}w_{0}}{qzb\left(2p_{0}-w_{0}\right)}$ can be obtained when $\frac{\partial \pi_{s}{}^{*}}{\partial \beta }=0$.  □

**Proposition** **9.** *Compared to the decentralized model led by sellers, agri-foods in the supply chain coordination model based on cost-sharing contracts are of higher quality and cheaper price under specific conditions.*

**Proof.**  It is known that $g^{*}-g_{2}^{*}=\frac{0.5k\beta w_{0}}{zbp_{0}\left(1-\beta \right)}>0$ and $\alpha^{*}-\alpha_{2}^{*}=\frac{2zb^{2}\left(1-\beta \right)p_{0}{}^{2}-zbq\left(1-\beta \right)p_{0}+zbqw_{0}\left(1-\beta \right)+0.5k^{2}w_{0}\beta }{2zb^{2}p_{0}{}^{2}\left(1-\beta \right)}$. Considering the numerator $2zb^{2}\left(1-\beta \right)p_{0}{}^{2}-zbq\left(1-\beta \right)p_{0}+zbqw_{0}\left(1-\beta \right)+0.5k^{2}w_{0}\beta$ as $\gamma \left(p_{0}\right)$, $\gamma \left(p_{0}\right)$ is thus a parabola with an opening upward about $p_{0}$. When $p_{0}=\frac{q}{4b}$, $\gamma \left(p_{0}\right)$ obtains the minimum value $Min\gamma \left(p_{0}\right)=-0.0625\left(1-\beta \right)zq^{2}+0.5zbqw_{0}\left(1-\beta \right)+0.25k^{2}w_{0}\beta$. Then, if $Min\gamma \left(p_{0}\right)\geq 0$, i.e., $w_{0}\geq \frac{0.25zq^{2}\left(1-\beta \right)}{2zbq\left(1-\beta \right)+k^{2}\beta }$, $\alpha^{*}\geq \;\alpha_{2}^{*}$ holds. Otherwise, when $0<w_{0}<\frac{0.25zq^{2}\left(1-\beta \right)}{2zbq\left(1-\beta \right)+k^{2}\beta }$, $\alpha^{*}<\alpha_{2}^{*}$ can be obtained.  □

From the equations $\pi_{n}^{d*}-\pi_{n}^{2*}=\frac{\left(0.5k^{2}w_{0}\left(1-0.5\beta \right)+\left(qbzw_{0}-qbzp_{0}-0.5k^{2}w_{0}{}^{2}\right)\left(1-\beta \right)\right)qw_{0}}{2\left(1-\beta \right)zb^{2}p_{0}{}^{2}}$ and $\pi_{s}^{d*}-\pi_{s}^{2*}=\frac{-{\left(qzb\left(\beta -1\right)\left(w_{0}-p_{0}\right)-0.5\beta k^{2}w_{0}\right)}^{2}+0.5\beta k^{4}w_{0}{}^{2}}{4z^{2}b^{3}p_{0}{}^{2}{\left(\beta -1\right)}^{2}}$, it can be seen that the positive or negative sign on the right side of the equation cannot be judged directly, which depends on the value of the relevant parameters. Therefore, the basic cost-sharing contract cannot significantly increase the profit of the participants.

Proposition 9 shows that the introduction of cost-sharing contracts based on the decentralized decision model can significantly improve the quality of products in the agricultural supply chain, and in most cases, the selling price of agricultural products will not be higher than that before the introduction of cost-sharing contracts. At this time, agri-foods in the supply chain based on cost-sharing contracts are of better quality and cheaper price. However, it cannot significantly improve the profit of agricultural supply chain participants.

## 6. Numerical Study

In this section, numerical examples are used to verify the above conclusions and carry out sensitivity analysis on the parameters in the model.

### 6.1. Numerical Example

According to the research on the agricultural market, the transaction price of several vegetables between cooperatives and supermarkets, the retail price of supermarkets, and other costs incurred in the agricultural–supermarket interface were considered comprehensively, and reasonable parameters q=30, $p_{0}=5$, $w_{0}=3$, $c_{n}=2$, $c_{s}=0.7$, k=1.2, b=0.8, and z=0.2 were set. Based on the results of the theoretical analysis, the optimal values of quality safety degree $g^{*}$, price subsidy level $\alpha^{*}$, market demand $d^{*}$, and total profit $\pi_{n}{}^{*}$, $\pi_{s}{}^{*}$, and $\pi_{\mathrm{ns}}{}^{*}$ of producers, sellers, and supply chain under different models are shown in Table 3.

As can be seen from Table 3, under this set of parameters, compared with the producer-dominated decentralized decision model, the quality of agri-foods is higher in the seller-dominated decision model, and the price compensation factor is also higher. The market sales volume does not change much. Although the profit of agricultural producers decreases slightly, the seller can obtain a higher profit, and the total profit of the supply chain increases.

The results of the algorithm can verify the conclusion of the above proposition. Obviously, the quality of agri-foods in the centralized model is the highest, followed by the Stackelberg model dominated by sellers, and finally the model dominated by agricultural producers. Although the actual market sales of centrally controlled cooperative game are not necessarily higher than those of non-cooperative game, the agricultural supply chain under the centralized game can still achieve more profits, i.e., the centralized decision model with the goal of maximizing the overall profit of the supply chain is more conducive to realizing the “quality and price” mechanism of agricultural supply.

### 6.2. Sensitivity Analysis

According to the assumptions above, *b* is the sensitivity of consumers to the price of agri-foods, while *k* is the sensitivity of consumers to the quality of agri-foods. By analyzing the sensitivity of *b* and *k*, the trend of the decision variables in the game model can be explored when the key parameters change.

#### 6.2.1. Sensitivity Analysis on Consumer Price Sensitivity Factors

**Figure 1:**
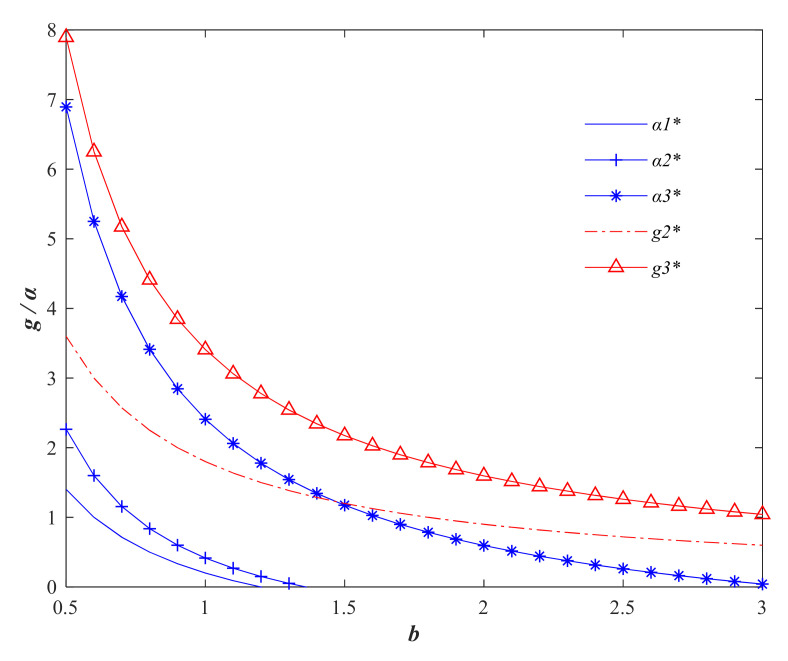
Variation of quality safety factor *g* and price compensation factor *α* with consumer price sensitivity factor *b*. (Variables with * are the optimal variable values).

Based on the parameters above, the variation of each variable in the three models regarding the consumer price sensitivity factor *b* is explored. From Figure 1, it can be seen that the price compensation factor α and the quality of agri-foods *g* decrease with the increase of consumer price sensitivity factor *b*. The trend indicates that when consumers become more and more sensitive to the price of agri-foods, the selling price of agri-foods will decrease with the increase of consumer price elasticity in order to increase sales, and at the same time, agricultural producers will choose to reduce quality improvement inputs to reduce costs. However, in general, the centralized decision with the goal of maximizing the overall benefit of the supply chain is still the optimal strategy to realize the quality and price of agri-foods.

**Figure 2:**
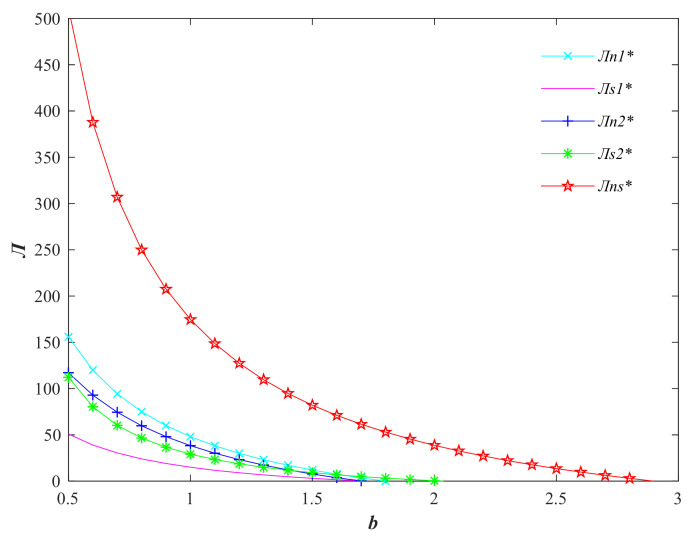
Variation of profits of agricultural supply chain participants with consumer price sensitivity factor *b*. (Variables with * are the optimal variable values).

From Figure 2, it can be seen that the profit of the participants in the agricultural supply chain decreases as the price elasticity of demand *b* increases, and the overall profit of the supply chain under the centralized decision is the largest. Since the profit of sellers is relatively small under this set of parameters, the overall profit distribution of sellers in the supply chain is slightly lower than that of producers. When the quality of agri-foods is promoted by the seller, the seller gains more profit compared with the producer; i.e., the profit is inclined to the dominant players in the supply chain.

#### 6.2.2. Sensitivity Analysis on Consumer Quality Sensitivity Factors

**Figure 3:**
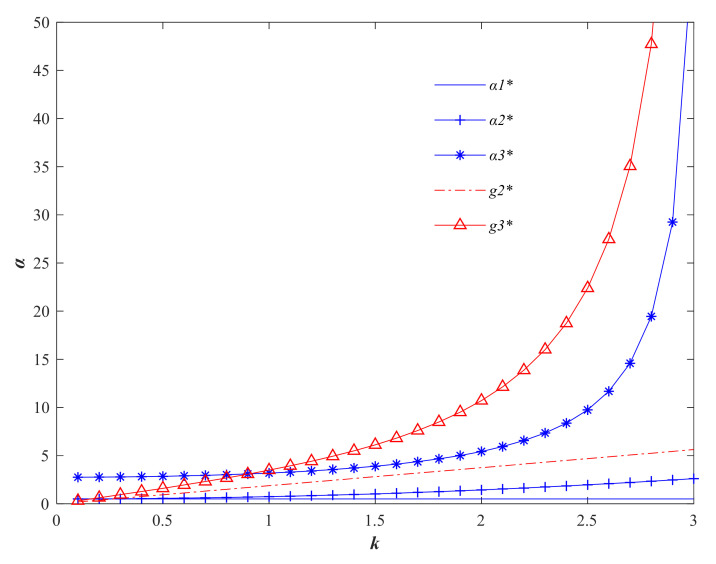
Variation of quality safety factor *g* and price compensation factor α with consumer quality sensitivity factor *k*. (Variables with * are the optimal variable values).

Using the above parameters, the variation of each variable in the three models with respect to the consumer quality sensitivity factor *k* is analyzed. From Figure 3, it can be seen that with the increase of *k*, the price compensation factor α and quality safety *g* in the producer-driven Stackelberg game model are at the lowest level and do not change. However, the price compensation factor α and quality safety degree *g* in the seller-dominated Stackelberg game model and the centralized decision model are both on the rise. In the producer-dominated Stackelberg game model, producers dominate the quality safety degree *g,* and sellers dominate the price compensation factor α. The trend shows that with consumers’ increasing care about the quality of agri-foods, producers are concerned that sellers will not compensate them for the cost of their efforts to improve quality. Therefore, in order to ensure their own maximum gain, producers will be stingy to increase the quality improvement input, which ultimately indicates that the producer is insensitive to the consumer’s quality demand elasticity. In Model 2 and 3, when consumers pay more and more attention on the quality of agri-foods, sellers will incentivize producers to improve the quality of agri-foods by increasing the price compensation factor.

**Figure 4:**
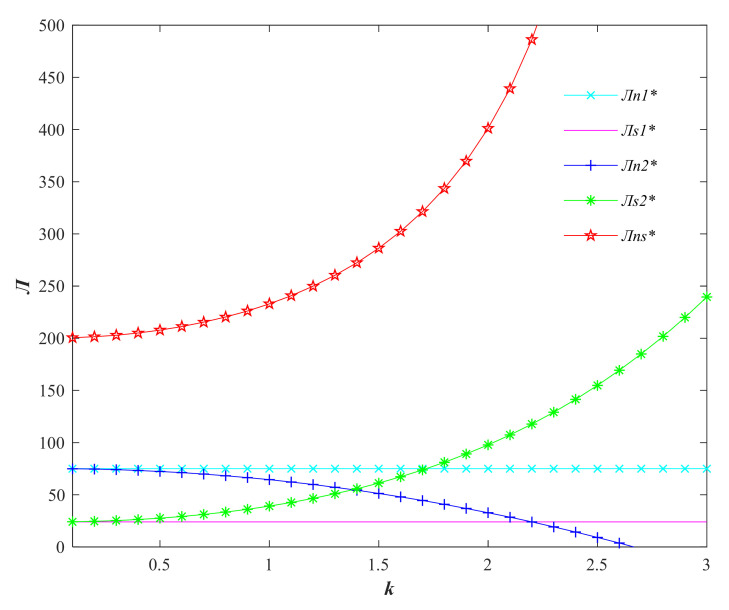
Variation of profit of agricultural supply chain participants with consumer quality sensitivity factor *k*. (Variables with * are the optimal variable values).

As can be seen in Figure 4, the producer is insensitive to the quality elasticity of demand because the producer does not actually make quality improvements in Model 1. The maximum profit of the seller in model 2 and the total profit of the supply chain in model 3 increase with the increase of the quality elasticity of demand, and the profit of the producer decreases with the increase of the quality elasticity of demand. When the quality elasticity of demand reaches a certain threshold, if the price increase is not large, the producers of agri-foods will be unprofitable due to the high quality input cost.

**Figure 5:**
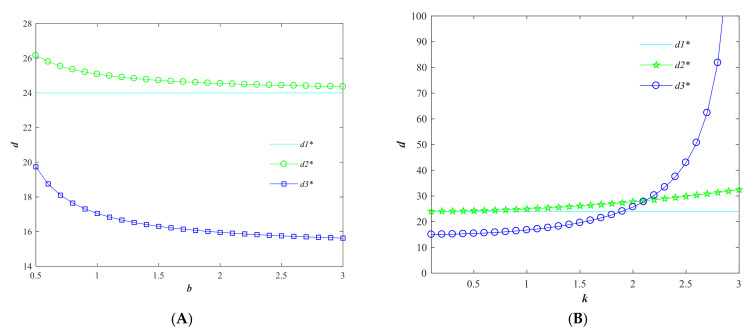
Variation of real market demand *d* with consumer price sensitivity factor *b* (**A**) and quality sensitivity factor *k* (**B**) (Variables with * are the optimal variable values).

From Figure 5, it can be seen that the actual market demand for agri-foods in models 2 and 3 decreases with the increase of consumer price sensitivity and diverges into two stages with the increase of quality sensitivity.

In the first stage, when the price sensitivity of consumers is very low, the actual market demand in the centralized decision model is the lowest, followed by the game model dominated by producers, and finally the game model dominated by sellers. The second stage is when the quality sensitivity of consumers reaches a certain value, the actual market demand in the centralized decision model starts to exceed the actual market demand in the decentralized decision model with the increase of quality sensitivity.

This indicates that when consumers are not sensitive to the quality of agri-foods, low-quality agri-foods sell better. When consumers are more and more concerned about the quality of agri-foods, the “high-quality and high-priced” produce sells better.

### 6.3. Analysis of Other Parameters

#### 6.3.1. The Impact of Agricultural Selling Prices on Profits

**Figure 6:**
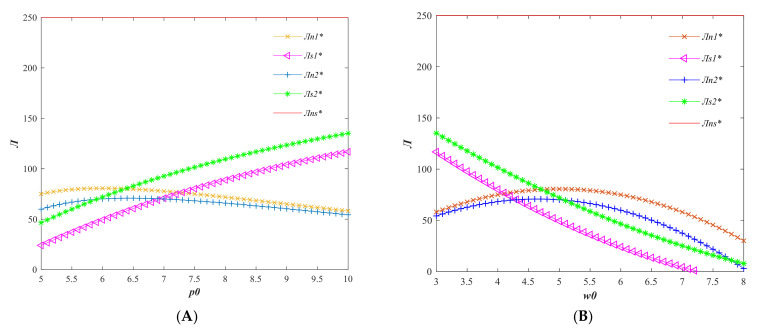
Variation of profits of supply chain participants with agricultural sales price $p_{0}$ (**A**) and wholesale price $w_{0}$ (**B**). (Variables with * are the optimal variable values).

The above parameters were also used to analyze the profit changes of supply chain participants when the retail price gradually increases from 5 to 10, and the results are shown in Figure 6A. As can be seen from the figure, the profit of sellers increases with the increase of selling price of agri-foods, while producers’ profit increases first and then decreases with the increase of selling price. With the increase of unit value of agri-foods, supply chain profit gradually inclines to sellers.

Considering that the profit of sellers in the original parameters is low, in order to observe the change of profit of supply chain participants with the wholesale price of agri-foods, the retail price is assumed to be 10. When the wholesale price increases from 3 to 8, the change of profit of supply chain participants is shown in Figure 6B. From the figure, it can be seen that with the increase of wholesale price, the profit of sellers gradually decreases, and the profit of producers first increases and then decreases. The total profit of the centralized decision supply chain is the largest, mainly related to parameters such as total regional sales volume rather than prices.

#### 6.3.2. The Impact of Cost-Sharing Factor

**Figure 7:**
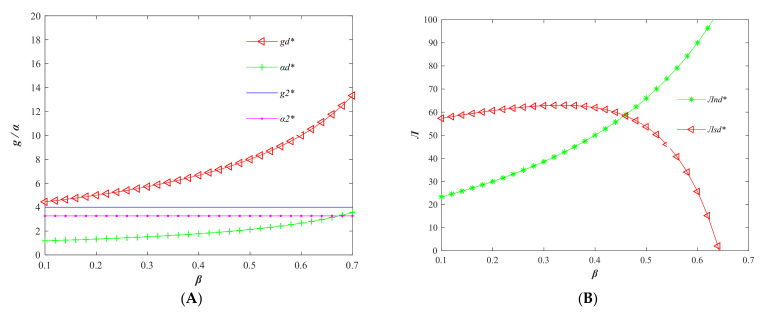
Variation of the main parameters (**A**,**B**) of the supply chain with the sharing ratio *β* after the introduction of the cost-sharing contract. (Variables with * are the optimal variable values).

According to Proposition 8, the parameters *k* = 1 and *b* = 0.375 are reset to observe the changes of some variables, such as the quality of agri-foods, the level of price subsidy, and the profit of supply chain participants with the sharing coefficient *β*, under the cost-sharing contract. The results are shown in Figure 7. It is easy to find from Figure 7A that the adoption of cost-sharing contracts can significantly improve the quality of agri-foods, and also the price subsidy level is lower than that before the introduction of cost-sharing contracts, which means that the price of agri-foods is more “high-quality and low-price” at this time. From Figure 7B, it can be seen that as the cost-sharing coefficient of sellers increases, the supply chain profit keeps shifting to producers. The producer and seller can achieve the same profit at a specific moment.

## 7. Discussion

From the analysis above, we find a very interesting phenomenon. That is, the decision of the quality improvement initiative depends on the decision-making style, the value of agri-foods, and the consumers’ consumption concept.

(1) In the decentralized decision-making model, the quality improvement of agri-foods led by sellers is better than that led by producers, but both are lower than the agricultural product quality level when the decision making is centralized to maximize the overall supply chain benefits. Therefore, from the consideration of improving the quality of agri-foods, it is the optimal strategy to improve the level of supply chain integration, establish supply chain alliances, and launch action plans of agri-food quality improvement jointly by supply chain members.
(2) Considering that the actual demand for agri-foods is affected by the wholesale price, the supply chain participants should choose decision-making strategies according to the characteristics of the products they operate when launching the action plan for agricultural produce quality improvement, from the perspective of opening up the market and increasing the market awareness of agricultural produce. Generally speaking, for general mass agri-foods, it is more favorable to expand sales volume by centralizing decisions for the supply chain to drive quality upgrades, while for high-end agri-foods with higher wholesale prices, it is more favorable to expand sales volume and open up the market for the supply chain to be led by sellers.
(3) The price and quality of agri-foods are affected by the price elasticity of demand and quality elasticity of demand. The more sensitive consumers are to price, the more popular low-quality and low-priced products will be, and the more sensitive consumers are to quality, the better quality and price mechanism is guaranteed when the overall benefit of the supply chain is maximized to improve the quality of agri-foods, and the quality improvement of agri-foods is more “good value for money” compared with the decentralized decision-making mode. Therefore, in order to promote the high-quality development of the agri-foods industry, raising the income level of agri-foods consumers and reducing the price elasticity of demand should be the first focus, and then, it is indispensable to promote the concept of food safety and improve the quality elasticity of demand so as to force the construction of a high-quality and high-quality price mechanism for agricultural produce.
(4) The use of cost-sharing contracts can significantly improve the quality of agri-foods in the supply chain, and the subsidized price of agri-foods under certain conditions is lower than before the introduction of the contract, which helps to reduce the selling price and increase sales. Moreover, cost-sharing contracts facilitate the transfer of profit distribution in the supply chain to the producers. Therefore, from the perspective of protecting “small farmers” and increasing consumer surplus, sellers should be encouraged to promote quality upgrading initiatives in the form of cost-sharing.

## 8. Conclusions

Three basic decision models involving the decision-making behavior on the quality improvement of agri-foods in the supply chain, namely, the decentralized decision model dominated by producers, the decentralized decision model dominated by sellers, and the centralized decision model, were constructed using game theory. The market demand of agri-foods depends on their sales price and supply quality is considered, and two decision variables of “quality safety” and “price compensation factor” are introduced into the linear inverse demand function for the first time. A cost-sharing contract is designed to study the relationship between product price, quality, and market demand in the supply chain. The results show that the centralized model is better than the two other models from the perspective of quality improvement. However, how to make decisions to achieve quality and price of agri-foods varies. Given that the study only analyzed the decisions within the supply chain, that is, the quality improvement action is voluntary and endogenous, future research can further discuss the influence of quality improvement on the behavior of other stakeholders and make clear how quality works in the market. Moreover, the participants in the agricultural supply chain are assumed to be risk-neutral. To validate the findings further, studies can be conducted with risk-appetitive and risk-averse participants.

## Figures and Tables

**Table 1:** The description of the notations.

Notations	Descriptions
$\pi_{n}$	Profit for the agricultural producer
$\pi_{s}$	Profit for the agricultural foods seller
$\pi_{ns}$	Overall profitability of the agricultural supply chain
*q*	Basic market demand, i.e., the order quantity determined by the seller based on previous years’ sales
*d*	Actual market demand, i.e., the amount of produce ultimately sold by the seller
$p_{0}$	Selling price of produce per unit for the seller before the quality improvement initiative
$w_{0}$	Selling price of produce per unit for the producer before the quality improvement initiative
$c_{n}$	Production cost per unit of agricultural produce
$c_{s}$	Cost of sales per unit of agricultural foods
*u*	Quality improvement inputs, i.e., the unit cost paid by producers to improve the quality of agricultural produce
*g*	Quality safety degree
α	Price compensation factor after quality inputs
*k*	Sensitivity coefficient of consumers to the quality of agricultural produce, i.e., quality elasticity of demand
*b*	Sensitivity coefficient of consumers to the price of agricultural produce, i.e., price elasticity of demand
*z*	Production impact factor

**Table 2:** Comparison of the three game models.

Variables	Model 1	Model 2	Model 3
$\alpha^{*}$	$\frac{q(p_{0}-w_{0})}{2bp_{0}^{2}}-1$	$\frac{qzb(p_{0}-w_{0})+0.5k^{2}w_{0}}{2zb^{2}p_{0}^{2}}-1$	$\frac{zq^{2}}{p_{0}(2bqz-k^{2})}-1$
$g^{*}$	0	$\frac{kw_{0}}{2zbp_{0}}$	$\frac{qk}{2bqz-k^{2}}$
$d^{*}$	$\frac{q(p_{0}+w_{0})}{2p_{0}}$	$\frac{qzb(p_{0}+w_{0})+0.5k^{2}w_{0}}{2zbp_{0}}$	$\frac{bzq^{2}}{2bqz-k^{2}}$
$\pi_{n}^{*}$	$\frac{q^{2}(p_{0}-w_{0})w_{0}}{2bp_{0}^{2}}-qc_{n}$	$\frac{(bqzp_{0}w_{0}-qzbw_{0}^{2}-0.25k^{2}w_{0}^{2})q}{2zb^{2}p_{0}^{2}}-qc_{n}$	
$\pi_{s}^{*}$	$\frac{q^{2}{\left(p_{0}-w_{0}\right)}^{2}}{4bp_{0}^{2}}-qc_{s}$	$\frac{q^{2}z^{2}{\left(p_{0}-w_{0}\right)}^{2}b^{2}+k^{2}qzbw_{0}(p_{0}-w_{0})+0.25k}{4z^{2}b^{3}p_{0}^{2}}-qc_{s}$	
$\pi_{ns}^{*}$			$\frac{0.5zq^{3}}{2bqz-k^{2}}-\left(c_{s}+c_{n}\right)q$

**Table 3:** Comparison of the three game models.

Variables	Model 1	Model 2	Model 3
$\alpha^{*}$	0.5	0.8	3.4
$g^{*}$	0	2.3	4.4
$d^{*}$	24	25.4	17.6
$\pi_{n}^{*}$	75	59.8	
$\pi_{s}^{*}$	24	46.5	
$\pi_{ns}^{*}$	99	105.3	249.9

Variables with * are the optimal variable values.

## Data Availability

Data is contained within the article.

## References

[1] Miao Y., Huang J., Liu R., Sun J. (2022). Research hotspots and development trend of high quality and high price in China: Visualization analysis based on citespace. Chin. J. Agric. Resour. Reg. Plan..

[2] Xu J., Yao G., Dai P. (2020). Quality Decision-Making Behavior of Bodies Participating in the Agri-Foods E-Supply Chain. Sustainability.

[3] Starbird S.A. (2005). Moral Hazard, Inspection Policy, and Food Safety. Am. J. Agric. Econ..

[4] Chen J. (2020). Game Analysis of Agri-food Quality Classification under Consumer Selection Behavior. Oper. Res. Manag. Sci..

[5] Messer K.D., Costanigro M., Kaiser H.M. (2017). Labeling food processes: The good, the bad and the ugly. Appl. Econ. Perspect. Policy.

[6] Bonroy O., Constantatos C. (2015). On the economics of labels: How their introduction affects the functioning of markets and the welfare of all participants. Am. J. Agric. Econ..

[7] Golan E., Kuchler F., Mitchell L., Greene C., Jessup A. (2001). Economics of food labeling. J. Consum. Policy.

[8] Hong X., Cao X., Gong Y., Chen W. (2020). Quality information acquisition and disclosure with green manufacturing in a closed-loop supply chain. Int. J. Prod. Econ..

[9] Li S., Shao L. (2018). Upgrade Strategies in The Presence of Strategic Consumers. Chin. J. Manag. Sci..

[10] Chen J., Liang L., Yao D.Q., Sun S. (2017). Price and quality decisions in dual-channel supply chains. Eur. J. Oper. Res..

[11] Li W., Chen J. (2018). Pricing and quality competition in a brand-differentiated supply chain. Int. J. Prod. Econ..

[12] Nie W., Bao H., Li T. (2021). A Theoretical Basis for and Path Towards Quality Grading System of Agricultural Products in China-Welfare Evaluation Based on Choice Experiment Survey. J. Agrotech. Econ..

[13] Nie W., Bao H., Li T. (2021). Blocking Mechanism and Attributes Selection of Consumers’ Searching Grading Information of Agricultural Products. J. Huazhong Agric. Univ..

[14] Shao Y., Chen G., Yang J. (2020). Potential Safety Hazard and Regulation Equilibrium of Primary Agricultural Products in Rural Areas. Collect. Essays Financ. Econ..

[15] Kang T., Mu Y. (2020). Asymmetry of Production and Marketing Information and Farmers’ Behavior of Fertilizer Overuse. J. Northwest A F Univ..

[16] Chen Z., Yuan B. (2020). Labor Price, Information Asymmetry and Investment in Fine Management Technology—From the Perspective of Differences in Quality Recognition Ability. J. Agrotech. Econ..

[17] Liu X., Dong Y. (2019). Quantity, Quality or Price-performance Ratio—On the Impetus and Transformation of China’s Agri-product Exports. J. Int. Trade.

[18] Abad P.L. (1994). Supplier pricing and lot sizing when demand is price sensitive. Eur. J. Oper. Res..

[19] Sadjadi S.J., Oroujee M., Aryanezhad M. (2005). Optimal production and marketing planning. Comput. Optim. Appl..

[20] Chan C.K., Kingsman B.G. (2007). Coordination in a single-vendor multi-buyer supply chain by synchronizing delivery and production cycles. Transp. Res. Part E.

[21] Dai T., Qi X. (2007). An acquisition policy for a multi-supplier system with a finite-time horizon. Comput. Oper. Res..

[22] SeyedEsfahani M.M., Biazaran M., Gharakhani M. (2011). A game theoretic approach to coordinate pricing and vertical co-op advertising in manufacturer-retailer supply chains. Eur. J. Oper. Res..

[23] Zhao Y., Wang S., Cheng T.E., Yang X., Huang Z. (2010). Coordination of supply chains by option contracts: A cooperative game theory approach. Eur. J. Oper. Res..

[24] Leng M., Parlar M. (2010). Game-theoretic analyses of decentralized assembly supply chains: Non-cooperative equilibriavs coordination with cost-sharing contracts. Eur. J. Oper. Res..

[25] Esmaeili M., Aryanezhad M.B., Zeephongsekul P. (2009). A game theory approach in seller-buyer supply chain. Eur. J. Oper. Res..

[26] Ren Y., He Z., Luning P.A. (2022). Luning, Performance of food safety management systems of Chinese food business operators in Tianjin. Food Control.

[27] Trienekens J., Zuurbier P. (2008). Quality and safety standards in the food industry, developments and challenges. Int. J. Prod. Econ..

[28] Yao G.X., Xu J. (2015). Agricultural supply chain decisions research under random yield. Ind. Eng. Manag..

[29] Aftab S., Yaseen M.R., Anwar S. (2017). Impact of rising food prices on consumer welfare in the most populous countries of South Asia. Int. J. Social Econ..

[30] Jiang S., Li S. (2015). Green Supply Chain Game Models and Revenue Sharing Contract with Product Green Degree. Chin. J. Manag. Sci..

[31] Yang Y., Yao G. Fresh agricultural products supply chain coordination considering consumers’ Dual preferences under carbon CAP-and-Trade mechanism. J. Ind. Manag. Optim..

